# Occult Cerebrovascular Disease and Late-Onset Epilepsy: Could Loss of Neurovascular Unit Integrity Be a Viable Model?

**DOI:** 10.1155/2011/130406

**Published:** 2011-03-06

**Authors:** Lorna M. Gibson, Stuart M. Allan, Laura M. Parkes, Hedley C. A. Emsley

**Affiliations:** ^1^Department of Acute Medicine, Western General Hospital, Edinburgh EH4 2XU, UK; ^2^Faculty of Life Sciences, University of Manchester, AV Hill Building, Oxford Road, Manchester M13 9PT, UK; ^3^School of Cancer and Imaging Sciences, University of Manchester, Stopford Building, Oxford Road, Manchester M13 9PT, UK; ^4^Department of Neurology, Royal Preston Hospital, Fulwood, Preston PR2 9HT, UK

## Abstract

Late-onset epilepsy (LOE) first occurs after 60 years of age and may be due to occult cerebrovascular disease (CVD) which confers an increased risk of stroke. However, patients with late-onset epilepsy are not currently consistently investigated or treated for cerebrovascular risk factors. We discuss how abnormalities of neurovascular unit function, namely, changes in regional cerebral blood flow and blood brain barrier disruption, may be caused by occult cerebrovascular disease but present clinically as late-onset epilepsy. We describe novel magnetic resonance imaging methods to detect abnormal neurovascular unit function in subjects with LOE and controls. We hypothesise that occult CVD may cause LOE as a result of neurovascular unit dysfunction.

## 1. Introduction

Late-onset epilepsy (LOE) is defined as epilepsy that first occurs after 60 years of age, and is considered by family doctors to be rare [1], despite the fact that it accounts for over a third of all incident epilepsy [2]. LOE occurs in approximately 4% of stroke patients [3], but importantly, LOE can present without a history of overt cerebrovascular disease (CVD), yet LOE confers a subsequent threefold increased risk of stroke [4]. It is widely assumed that LOE is often attributable to otherwise occult CVD. However, at least in the UK, patients with LOE tend to be prescribed anticonvulsant medication, but the opportunity that LOE presents as a marker of increased stroke risk may be lost if a presentation of LOE does not prompt clinicians to screen for other vascular risk factors and initiate appropriate vascular secondary prevention measures, for which there is a strong case [4, 5]. 

Occult CVD may be detected on brain imaging but by definition does not manifest otherwise clinically. Structural imaging markers of occult CVD are thought to include cortical or subcortical infarcts, white matter hyperintensities, leukoaraiosis (LA), cerebral atrophy, and brain microbleeds (BMBs) which are a marker particularly of cerebral microangiopathy, and strongly associated with hypertension [6]. However, between 72 and 94% of occult infarcts are subcortical, yet epilepsy derives from the cortex. If occult CVD is aetiologically important in LOE then markers of CVD would be expected to have a more diffuse anatomical distribution than described previously. Subcortical lesions in isolation would not be expected to cause the disruption of corticocortical or subcorticocortical circuits that would be a necessary substrate for epileptogenesis. Markers of functional rather than structural integrity may then be necessary to resolve this apparent discrepancy. For example, a corollary is seen in patients with frontal lobe cognitive dysfunction where structural lesions on MRI are confined anatomically to subcortical regions. In this group, frontal lobe hypometabolism measured using FDG-PET correlated with subcortical lacunes and white matter lesions [7]. We hypothesise that occult CVD may cause LOE via neurovascular unit dysfunction, namely, producing changes in cerebral blood flow (CBF) and/or disruption of the blood brain barrier (BBB). We present evidence for and against this hypothesis and describe novel MRI methods for detecting neurovascular unit dysfunction in subjects with LOE and control participants. 

## 2. The Neurovascular Unit and Neurovascular Coupling

The structural and functional integrity of the central nervous system depends on coupling between neural activity and (CBF), and regulation of transport across the (BBB). These two critical processes rely on the coordinated activity of a “neurovascular unit” comprising the cerebral endothelium, neurones, and glial cells. In the normal healthy state the increase in CBF produced by brain activity, termed functional hyperaemia, is an example of the close interaction between the neurones, glia, and vascular cells. Neurovascular coupling (NVC) can be defined as the relationship between the neural response which can be measured by a variety of methods and the associated vascular response, that is, change in CBF. 

## 3. Assessing Neurovascular Unit Function

Functional MRI can help assess the functional capacity of cerebral blood vessels to respond to neuronal activation [8], using the Blood Oxygen Level Dependent (BOLD) signal. However, the BOLD signal is physiologically complex, depending on the relative change in CBF and oxygen metabolism (CMRO2). A relatively new technique using simultaneous arterial spin labelling (ASL) and BOLD allows quantification of these component parts. 

ASL provides a quantitative measurement of CBF [9]. The BOLD signal is sensitive to deoxyhaemoglobin and is dependent on changes to CBF and CMRO2, which both change during neuronal activity [10]. A calibrating procedure is employed, which may consist of asking patients to hold their breath to induce hypercapnia (excess carbon dioxide in the blood). This causes CBF to increase and it is assumed that CMRO2 does not change, so isolating the CBF component of the BOLD signal. Using simultaneous BOLD and ASL imaging allows the calculation of a calibration factor which relates BOLD signal change to changes in CBF [8]. Patients then perform a task which increases cortical neuronal activity and oxygen demand. CBF change is measured using ASL again, and the calculated calibration factor enables CMRO2 to be calculated from the BOLD signal change [8]. Measurements of CBF can then be compared with CMRO2 during the task. It may be possible to see that CMRO2 becomes uncoupled from CBF which may indicate that the blood vessels cannot deliver the increases in blood flow required. This reduction in functional capacity may suggest occult ischaemia.

Studies show an association between reduced regional CBF (rCBF) and occult infarcts. In a study of 246 clinically neurologically normal patients, 32 (13.0%) patients were found to have occult lacunar infarcts. Of the 34 patients aged over 60, 7 (20.6%) were found to have occult lacunar infarcts. Patients with occult lacunar infarcts have significantly reduced rCBF in both right and left frontotemporal regions compared to patients without occult lacunar infarcts (*P* < .05) [11]. Furthermore nine patients (mean age 63.4, range 58–67) with occult infarcts were found to have reduced rCBF in all cortices (significant in the temporal and parietal cortices, *P* < .05) and a nonsignificant reduction in rCMRO2 globally compared to nine controls without occult infarcts, however, patients with epilepsy were not specifically excluded [12]. These findings suggest a diffuse vascular encephalopathy, which may result in a reduced functional capacity of the blood vessels and subsequent ischaemia.

Patients with both LOE and LA have significantly reduced regional CMRO2 (rCMRO2) in all cortices (frontal, temporal, parietal, and occipital) compared to controls, indicating hypometabolism in these regions (*P* < .05) [13]. These patients also have significantly reduced rCBF in all cortices compared to controls (*P* < .05) [13]. However, these results may have been confounded as no measure of carotid artery stenosis was made, which affects CBF measurements [14]. Reduced rCBF and rCMRO2 may indicate either a degenerative or vascular underlying process [13]. If the cortical neurones are degenerating their demand for oxygen reduces resulting in hypometabolism and reduced blood flow. On the other hand, damage to cortical blood vessels may result in reduced blood flow, therefore reducing oxygen delivery to the tissues and inducing hypometabolism. Both vascular and degenerative processes may be present in older patients. 

## 4. A Preliminary Investigation of Neurovascular Coupling in Patients with Late-Onset Epilepsy

With relevant ethical and research governance approvals, we undertook an initial pilot clinical study in subjects with LOE and conventional vascular risk factors (aged 60 or over at onset, with at least 2 seizures of presumed partial onset), and control subjects with (RF+) or without (RF−) vascular risk factors. We excluded subjects with a history of clinically overt transient ischaemic attack or stroke, focal motor or sensory signs, known aetiology for epilepsy or acute symptomatic seizures, cognitive dysfunction sufficient to interfere with daily activities, or any other active, significant medical condition likely to complicate assessment.

**Figure 1:**
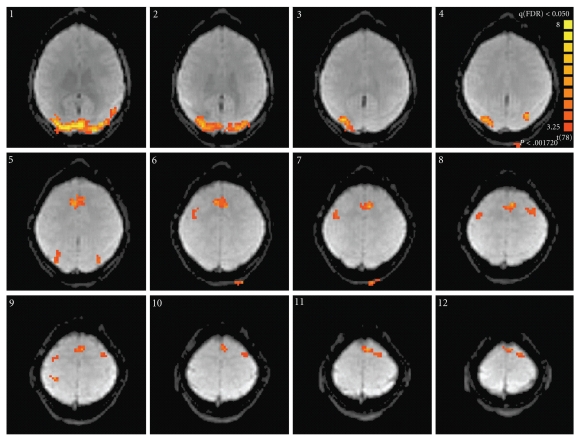
Regions of activation for the combined motor and visual task in a healthy volunteer, presented at a false detection rate of *P* < .05.

Six participants were scanned using a 3T MRI scanner (Siemens Trio, Erlangen, Germany). The body coil was used for signal transmission and an 8-channel phased array head coil was used for signal collection. The MRI protocol comprised precerebral extracranial and intracranial time of flight angiography, high resolution T1 and T2 weighted images, T2* and fluid attenuated inversion recovery (FLAIR) anatomic images, and a pulsed arterial spin labelling (ASL) sequence (Q2TIPS). Simultaneous blood oxygenation level dependent (BOLD) contrast functional MRI (fMRI) was also performed, with the participant performing a breath-hold paradigm (6 cycles of breath holding on inspiration for 15 s followed by normal breathing for 40 s) and a combined visual and motor paradigm (8 cycles of bilateral hand squeezing of foam pads for 20 s followed by 20 s rest, cued with a flashing visual display). Custom written MATLAB programs (The MathWorks Inc., MA) were used to generate CBF and BOLD images using an appropriate kinetic model [9]. Standard analysis within BrainVoyager was used to identify regions that were significantly active (false detection rate *P* < .05) during the combined visual and motor task (Figure 1). CBF and BOLD time courses during both the motor task and breath hold were recorded within these regions using the model previously described [8].

**Figure 2:**
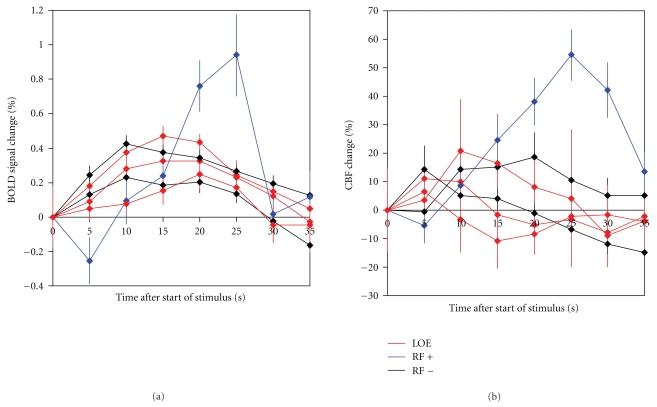
BOLD (a) and CBF (b) responses in participants' visual cortices. CBF is measured in mL blood/min/100 g tissue. Times are in seconds from the start of the motor task, that is, visually-cued hand-squeezing began at time 0 and lasted 20 seconds, followed by 20 seconds of rest. Error bars represent the standard error over the 8 cycles.

Data were available for 3 subjects with LOE, and 3 controls (1 RF+, 2 RF-). 1 patient and 1 control (RF+) had mild white matter hyperintensities; all LOE subjects and 1 control (RF+) had reduced cortical volume compared to the other 2 controls (RF-); no other structural lesions were identified. Only one participant had extracranial or intracranial stenosis (RF+ control, 40% stenosis at origin of right internal carotid artery). Average whole brain (mean 35.5 mL blood/min/100 g tissue, range 30.3 to 39.3), grey matter (mean 49.6, range 42.6 to 55.3) and white matter (mean 21.0) perfusion was similar across all participants. During the combined visual and motor task, all subjects showed significant activation in the visual cortex, whereas motor cortex activation was more variable, presumably due to different performance levels on the task. Therefore, responses were recorded from the visual cortex only. BOLD and CBF responses to breath hold were very variable, to the extent that they could not be used reliably to calibrate the BOLD signal during activation. Future work should consider the use of gas-induced hypercapnia [15] or hyperoxia [16, 17] for calibration as, while they are more difficult to administer, they are better controlled than breath hold [18]. The BOLD and CBF responses showed differences in timing between the groups, with the RF+ control and the LOE subjects showing a later peak in the BOLD response compared to RF−controls (Figure 2). The finding of reduced cortical volumes among LOE subjects and the RF+ control compared to RF−controls probably reflects otherwise occult cerebrovascular disease given these participants' vascular risk factor burden. 

This initial work demonstrates the feasibility of simultaneous ASL perfusion and BOLD measures in LOE patients, albeit with limited interpretation possible in this pilot study. Further work will be needed to explore potential impairment of NVC in LOE in addition to the apparent structural changes probably attributable to otherwise occult cerebrovascular disease. 

## 5. Neurovascular Coupling and Epilepsy

Epilepsy is an abnormal state which places supranormal demands on the cerebral autoregulatory mechanisms consequent upon an enormous increase in CMRO2 following interictal and ictal events [19]. Normal NVC mechanisms may not be relevant to the epileptic brain. It has been long debated whether or not CBF is adequate to meet the increased metabolic demands of epilepsy. Originally, based on histological similarities between ischaemic and epileptic brain damage, it was proposed that the cell damage following status epilepticus was caused by cerebral hypoxia. However, subsequent work refuted this theory, finding a greater increase in CBF relative to CMRO2, that cellular damage differs between status epilepticus and hypoxic injury, that seizures induce increases rather than reduction in venous oxygenation, and evidence of oxidation in the mitochondrial transport chain, NADH and cytochrome oxidase, increases in tissue pO2, and evidence of tissue injury in the absence of hypoxia [19]. 

## 6. How Might Loss of NVU Integrity in CVD Be Linked to Epileptogenesis?

**Figure 3:**
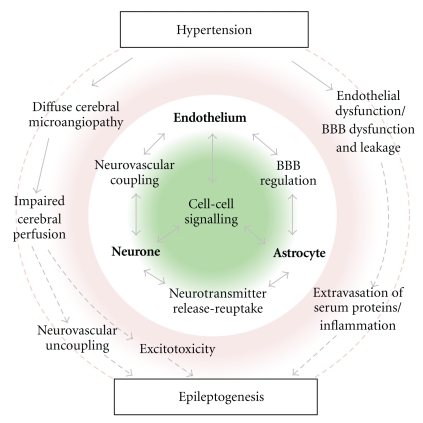
Proposed model of epileptogenicity in occult CVD. The central green/white circle depicts the elements of the healthy neurovascular unit. The outer red/white circle depicts possible sequelae of hypertension, a key determinant of CVD. Structural and functional integrity of the neurovascular unit may be compromised by a diffuse cerebral microangiopathy and endothelial and BBB dysfunction with BBB leakage. Potential mechanisms leading to epileptogenesis might then include impaired cerebral perfusion and neurovascular uncoupling, excitotoxicity occurring as a consequence of ischaemia, or extravasation of serum proteins and inflammation.

There are conceivably several mechanisms linking disruption of NVU integrity—in terms of either an altered relationship between neural activity and CBF, or dysregulation of transport across the BBB—and epileptogenesis in the context of CVD (Figure 3). Cerebral amyloid angiopathy (CAA), a neurovascular degenerative disorder resulting in progressive neurovascular unit dysfunction and in some cases associated with seizures, is briefly discussed as a potentially useful case study in this area. 

### 6.1. Changes in CBF

The notion that haemodynamic alterations might localise and predict the onset of seizures is not new but advances in imaging, including BOLD fMRI, has refocused attention on this area. Recently, changes in BOLD signal have been observed to precede scalp EEG epileptic spikes, raising the possibility that BOLD signal change is due to an event “invisible” to the scalp EEG, or that an abrupt haemodynamic change is responsible for the epileptic discharge [20]. However, whether it is hypoperfusion, hyperperfusion, or indeed the transition between the two states that might theoretically be important is not straightforward to determine. A study of 40 patients with complex partial seizures using 133Xe CT imaging at rest and during a light stimulation procedure found a significant increase in rCBF in the region of the suspected epileptic focus in nonlesional patients [21]. Middle cerebral artery blood flow velocity assessed with transcranial Doppler ultrasonography during simultaneous EEG recording, reveals asymmetric perfusion increases closely related to onset and cessation of EEG seizure activity during simple partial motor seizures [22]. Another study using perfusion CT to measure rCBF with EEG correlation found increased rCBF during subtle status epilepticus but regional hypoperfusion in postictal patients [23]. Indeed, postictal (or Todd's) paresis has been found, using perfusion MRI, to be accompanied by a reversible global hemispheric hypoperfusion, indicating transient but marked cerebrovascular dysfunction in postictal paresis [24]. In this instance, discrimination from emerging stroke is clearly important to avoid potentially harmful interventions such as thrombolysis. The occurrence of early onset seizures after acute ischaemic stroke has been related to the severity of stroke, with thrombolysis arguably reducing the occurrence of late-onset seizures after stroke, perhaps through improved reperfusion of the ischaemic brain regions [25]. Intriguingly, seizures occurring during thrombolytic therapy for acute ischaemic stroke and heralding dramatic recovery have been attributed to cerebral reperfusion and/or hyperperfusion [26]. 

An experimental model of stroke-induced epilepsy may shed light on how reduced rCBF producing ischaemia leads to LOE. The model uses glutamate excitotoxic injury to produce an infarct surrounded by an ischaemic penumbra. The ischaemic neurones survive the glutamate injury and produce spontaneous recurrent epileptiform discharges [27] which may be due to their increased basal levels of intracellular calcium [28]. However, this model uses 2-day-old rats and may not be appropriate for modelling LOE in occult CVD. An adult animal model of occult CVD is therefore needed to investigate the mechanisms of epileptogenesis which may be more applicable to LOE patients. 

### 6.2. BBB Dysfunction

The microvasculature is abnormal in cerebral small vessel disease (cSVD), with loss of smooth muscle cells, vessel wall thickening, luminal narrowing, and increased vessel stiffness. These changes may contribute to the attenuation of vasomotor reactivity in response to hypercapnia or acetazolamide seen in cSVD [29], and consequently lead to an impairment of NVC. Such impairment of NVC appears to be specific for cSVD [30]. Recently BBB breakdown has received considerable attention as a cause of cSVD. BBB breakdown may lead to ischaemia, LA and lacunar infarcts [31]. Mild chronic hypertension may damage cerebrovascular endothelium in small vessels leading to thickening of the arterial wall and narrowing of the lumen, resulting in ischaemia (LA if ischaemia occurs in white matter) or infarction. Alternatively, the damage to the arterial wall may progress, causing disintegration and a leak of blood [31]. BBB leak may be visualised as small areas of low signal on T2*-weighted MR images which may be deposits of haemosiderin, a breakdown product of haemoglobin, usually indicating a BMB [32].

Seiffert and colleagues created a model of BBB breakdown-induced epilepsy by perfusing a section of adult rat somatosensory cortex with serum [33]. 77% of slices from treated cortices developed increased excitability compared to sham-operated controls. As the changes in firing rate were only evident four days after treatment, the authors suggest that an accumulation of albumin in the extracellular space due to BBB leak is required for epileptogenesis. More recent work suggests that BBB breakdown may lead to changes in astrocyte gene expression in astrocytes, which predicts impaired uptake of extracellular potassium [34]. The increased extracellular K+ may facilitate the conduction of excessive neuronal discharges, resulting in epilepsy [35]. However, the results of these animal experiments may not be applicable to human patients with epilepsy. Furthermore, to determine whether BBB breakdown in this model is truly occult, the motor cortex could be perfused with serum and the animals' motor function observed.

Novel MRI techniques to assess BBB opening could complement functional MRI to offer a fascinating insight into neurovascular unit function in terms of metabolism, neurovascular response, and BBB integrity. For example, there is increasing interest in the use of dynamic contrast-enhanced MRI in a range of conditions, including ageing and cerebral microvascular disease [36]. The prospect of applying this technique in patients with LOE is appealing. 

### 6.3. Cerebral Amyloid Angiopathy: A Case Study in Neurovascular Unit Disruption and Epileptogenesis

Cerebral amyloid angiopathy (CAA) is a disorder caused by the accumulation of amyloid in cerebral vessels which leads to progressive dysfunction of the neurovascular unit, failure of vascular reactivity, smooth muscle cell loss, and eventual breakdown of vessel integrity [37]. CAA is associated with BMBs and recurrent lobar intracerebral haemorrhage. It is an increasingly recognised disorder in the elderly and can be associated with recurrent seizures. Other manifestations include white matter disease, cortical infarcts, and cognitive dysfunction. Transient neurological events characterised by spread of symptoms to contiguous body areas during episodes, in keeping with seizures, have been described in CAA [38]. Seizures have also been reported in CAA by other workers [39]. CAA-associated vascular inflammation has also been described in a subset of CAA patients presenting with cognitive decline and seizures [40]. Further studies of neurovascular unit dysfunction in CAA and the potential relationship with epileptogenesis may prove valuable. 

## 7. Conclusion

Occult CVD may cause LOE via neurovascular unit dysfunction, namely, producing changes in (CBF) and/or disruption of the (BBB). We have demonstrated the feasibility of a novel MRI technique to investigate NVC, reflecting underlying neurovascular unit integrity, in this population. Further studies will be important given the likely increasing burden of CVD and its complications in an ageing population. Increasing recognition of entities such as CAA is likely to provide further opportunities for research in this area. At the current time, conventional imaging techniques remain the mainstay of the identification of otherwise occult CVD, and it is evidence such as this that should prompt screening for other vascular risk factors and the initiation of appropriate vascular secondary prevention measures. 

## References

[1] Craig I, Tallis RC (1991). General practitioner management of adult-onset epilepsyanalysed. *Care Elderly*.

[2] Tallis R, Hall G, Craig I, Dean A (1991). How common are epileptic seizures in old age?. *Age and Ageing*.

[3] Burn J, Dennis M, Bamford J, Sandercock P, Wade D, Warlow C (1997). Epileptic seizures after a first stroke: the Oxfordshire community stroke project. *British Medical Journal*.

[4] Cleary P, Shorvon S, Tallis R (2004). Late-onset seizures as a predictor of subsequent stroke. *The Lancet*.

[5] Sudlow CLM (2004). Epilepsy and stroke. *The Lancet*.

[6] Cordonnier C, Al-Shahi Salman R, Wardlaw J (2007). Spontaneous brain microbleeds: systematic review, subgroup analyses and standards for study design and reporting. *Brain*.

[7] Reed BR, Eberling JL, Mungas D, Weiner M, Kramer JH, Jagust WJ (2004). Effects of white matter lesions and lacunes on cortical function. *Archives of Neurology*.

[8] Buxton RB, Uludağ K, Dubowitz DJ, Liu TT (2004). Modeling the hemodynamic response to brain activation. *NeuroImage*.

[9] Parkes LM, Tofts PS (2002). Improved accuracy of human cerebral blood perfusion measurements using arterial spin labeling: accounting for capillary water permeability. *Magnetic Resonance in Medicine*.

[10] Jezzard P, Buxton RB (2006). The clinical potential of functional magnetic resonace imaging. *Journal of Magnetic Resonance Imaging*.

[11] Kobayashi S, Okada K, Yamashita K (1991). Incidence of silent lacunar lesion in normal adults and its relation to cerebral blood flow and risk factors. *Stroke*.

[12] Nakane H, Ibayashi S, Fujii K (1998). Cerebral blood flow and metabolism in patients with silent brain infarction: occult misery perfusion in the cerebral cortex. *Journal of Neurology Neurosurgery and Psychiatry*.

[13] De Reuck J, Decoo D, Boon P, Strijckmans K, Goethals P, Lemahieu I (1996). Late-onset epileptic seizures in patients with leukoaraiosis: a positron emission tomographic study. *European Neurology*.

[14] Trivedi RA, Green HAL, U-King-Im J (2005). Cerebral haemodynamic disturbances in patients with moderate carotid artery stenosis. *European Journal of Vascular and Endovascular Surgery*.

[15] Davis TL, Kwong KK, Weisskoff RM, Rosen BR (1998). Calibrated functional MRI: mapping the dynamics of oxidative metabolism. *Proceedings of the National Academy of Sciences of the United States of America*.

[16] Chiarelli PA, Bulte DP, Wise R, Gallichan D, Jezzard P (2007). A calibration method for quantitative BOLD fMRI based on hyperoxia. *NeuroImage*.

[17] Goodwin JA, Vidyasagar R, Balanos GM, Bulte D, Parkes LM (2009). Quantitative fMRI using hyperoxia calibration: reproducibility during a cognitive Stroop task. *NeuroImage*.

[18] Bulte DP, Drescher K, Jezzard P (2009). Comparison of hypercapnia-based calibration techniques for measurement of cerebral oxygen metabolism with MRI. *Magnetic Resonance in Medicine*.

[19] Schwartz TH (2007). Neurovascular coupling and epilepsy: hemodynamic markers forlocalizing and predicting seizure onset. *Epilepsy Current*.

[20] Hawco CS, Bagshaw AP, Lu Y, Dubeau F, Gotman J (2007). BOLD changes occur prior to epileptic spikes seen on scalp EEG. *NeuroImage*.

[21] Valmier J, Touchon J, Baldy-Moulinier M (1989). Interictal regional cerebral blood flow during non specific activation test in partial epilepsy. *Journal of Neurology Neurosurgery and Psychiatry*.

[22] Niehaus L, Wieshmann UC, Meyer BU (2000). Changes in cerebral hemodynamics during simple partial motor seizures. *European Neurology*.

[23] Wiest R, von Bredow F, Schindler K (2006). Detection of regional blood perfusion changes in epileptic seizures with dynamic brain perfusion CT-A pilot study. *Epilepsy Research*.

[24] Rupprecht S, Schwab M, Fitzek C, Witte OW, Terborg C, Hagemann G (2010). Hemispheric hypoperfusion in postictal paresis mimics early brain ischemia. *Epilepsy Research*.

[25] De Reuck J, Van Maele G (2010). Acute ischemic stroke treatment and the occurrence of seizures. *Clinical Neurology and Neurosurgery*.

[26] Rodan LH, Aviv RI, Sahlas DJ, Murray BJ, Gladstone JP, Gladstone DJ (2006). Seizures during stroke thrombolysis heralding dramatic neurologic recovery. *Neurology*.

[27] Sun DA, Sombati S, DeLorenzo RJ (2001). Glutamate injury-induced epileptogenesis in hippocampal neurons: an in vitro model of stroke-induced "epilepsy". *Stroke*.

[28] Sun DA, Sombati S, Blair RE, DeLorenzo RJ (2004). Long-lasting alterations in neuronal calcium homeostasis in an in vitro model of stroke-induced epilepsy. *Cell Calcium*.

[29] Terborg C, Gora F, Weiller C, Röther J (2000). Reduced vasomotor reactivity in cerebral microangiopathy: a study with near-infrared spectroscopy and transcranial Doppler sonography. *Stroke*.

[30] Schroeter ML, Cutini S, Wahl MM, Scheid R, Yves von Cramon D (2007). Neurovascular coupling is impaired in cerebral microangiopathy—an event-related Stroop study. *NeuroImage*.

[31] Wardlaw JM, Sandercock PAG, Dennis MS, Starr J (2003). Is breakdown of the blood-brain barrier responsible for lacunar stroke, leukoaraiosis, and dementia?. *Stroke*.

[32] Roob G, Fazekas F (2000). Magnetic resonance imaging of cerebral microbleeds. *Current Opinion in Neurology*.

[33] Seiffert E, Dreier JP, Ivens S (2004). Lasting blood-brain barrier disruption induces epileptic focus in the rat somatosensory cortex. *Journal of Neuroscience*.

[34] David Y, Cacheaux LP, Ivens S (2009). Astrocytic dysfunction in epileptogenesis: consequence of altered potassium and glutamate homeostasis?. *Journal of Neuroscience*.

[35] Heinemann U, Gabriel S, Jauch R (2000). Alterations of glial cell function in temporal lobe epilepsy. *Epilepsia*.

[36] Armitage PA, Farrall AJ, Carpenter TK, Doubal FN, Wardlaw JM Use of dynamic contrast-enhanced MRI to measure subtle blood-brain barrier abnormalities.

[37] Zipfel GJ, Han H, Ford AL, Lee JM (2009). Cerebral amyloid angiopathy progressive disruption of the neurovascular unit. *Stroke*.

[38] Greenberg SM, Vonsattel JPG, Stakes JW, Gruber M, Finklestein SP (1993). The clinical spectrum of cerebral amyloid angiopathy: presentations without lobar hemorrhage. *Neurology*.

[39] Karabatsou K, Lecky BRF, Rainov NG, Broome JC, White RP (2007). Cerebral amyloid angiopathy with symptomatic or occult subarachnoid haemorrhage [1]. *European Neurology*.

[40] Kinnecom C, Lev MH, Wendell L (2007). Course of cerebral amyloid angiopathy-related inflammation. *Neurology*.

[41] Gibson LM, Parkes LM, Emsley HCA Occult cerebrovascular disease in late-onset epilepsy: a literature review and novel hypothesis.

[42] Gibson LM, Parkes LM, Emsley HCA (2008). Occult cerebrovascular disease in late-onset epilepsy: a literature review and novel hypothesis. *Journal of Neurology*.

